# Optimizing glucocorticoid therapy for Behçet's uveitis: efficacy, adverse effects, and advances in combination approaches

**DOI:** 10.1007/s10792-023-02808-w

**Published:** 2023-07-07

**Authors:** Biao Li, Haoran Li, Qun Huang, Yanlin Zheng

**Affiliations:** Hospital of Chengdu University of Traditional Chinese Medicine, Chengdu, China

**Keywords:** Behçet's syndrome, Uveitis, Glucocorticoids, Treatment

## Abstract

Behçet's uveitis (BU) is a debilitating manifestation of Behçet's disease, often requiring prompt and aggressive treatment to prevent vision loss. Glucocorticoids (GCS) serve as a first-line therapy for BU; however, their long-term, high-dose use can result in significant adverse effects. This review summarizes the efficacy, adverse effects, and advances in combination therapy involving GCS for the management of BU. We discuss the benefits and drawbacks of various GCS administration routes, including periocular and intravitreal injections, intravitreal sustained-release devices, and systemic therapy, highlighting the role of fluocinolone acetonide and dexamethasone as primary sustained-release formulations. Moreover, we underscore the importance of combining GCS with immunosuppressive drugs and biological agents to minimize adverse reactions and optimize therapeutic outcomes. The review concludes that, while GCS remain a crucial component of BU treatment, careful consideration of their administration and combination with other therapies is essential to achieve long-term remission and improved visual outcomes for patients with BU.

## Introduction

Behçet's Syndrome (BS) constitutes a relapsing immune-inflammatory disorder hallmarked by occlusive vasculitis and neutrophil hyperfunction as fundamental pathologic alterations [1, 2]. The disease manifests as a triad of oral ulcers, genital ulcers, and non-infectious uveitis [2], while also involving multiple organs and systems, such as blood vessels, skin, gastrointestinal tract, and nervous system [3].

BS occurs worldwide, with higher prevalence in regions along the ancient Silk Route, particularly in the Middle East, the Mediterranean basin, and the Far East [4]. Ocular involvement, often bilateral, affects an estimated 35–90% of BS patients [5, 6] and is more frequently observed in male patients with a younger age of disease onset, who are at a higher risk of visual loss [7]. BS typically affects young adults between the ages of 25 and 35 [8], with ocular involvement commonly presenting 2–4 years after disease onset, and is the leading cause of non-infectious uveitis in referral centers in Italy [9] and Turkey [10].

BS frequently presents with Behçet's uveitis (BU) [11, 12], a particularly challenging uveitis subtype characterized by a dismal prognosis and an elevated risk of blindness compared to other prevalent types such as Vogt-Koyanagi-Harada (VKH) [13–16]. BU incidence exhibits marked regional variability but affects a considerable proportion of BS patients [2, 17, 18], typified by recurrent episodes and protracted non-healing, culminating in bilateral panuveitis and irreversible visual impairment for the majority of patients [15, 19–21]. The alarming incidence of blindness in BU patients significantly impairs the quality of life for affected individuals [5, 13, 14].

BU pathogenesis remains incompletely elucidated, with the prevailing hypothesis implicating an autoimmune inflammatory response mediated by neutrophils and Th1 cells [22], resulting in non-granulomatous uveitis and retinal vasculitis [5, 23]. Consequently, precise BU treatment poses a formidable challenge, with no standard treatment protocol established [24]. The primary treatment objective encompasses achieving and sustaining remission by attenuating the immune response, controlling inflammation, and averting severe complications such as vision loss. Glucocorticoids (GCS), immunosuppressants, and biologics currently serve as treatment options for BU [25–27]. It is important to highlight from the outset that, for patients with Behçet's Uveitis (BU) involving the posterior segment of the eye, systemic corticosteroid monotherapy is contraindicated. In these cases, long-term disease control is typically achieved through the use of conventional immunosuppressive agents or biologics. Local corticosteroid injections or implants can provide valuable adjunctive support, but should not be relied upon as the primary mode of treatment. This nuanced understanding of the therapeutic approach forms the basis for the ensuing review of glucocorticoid therapy optimization in the management of BU. This review seeks to offer a synopsis of the research advancements concerning GCS in BU treatment over the past decade.

Since their initial application in uveitis treatment in 1950 [28, 29], GCS have emerged as the foremost therapeutic option for BU, displaying potent anti-inflammatory and immunosuppressive effects while modulating the metabolism of numerous nutrients and the functional activities of diverse organ systems. Currently, various GCS formulations exist, which can be classified according to their administration route as either local or systemic. Local administration encompasses topical eye drops, periocular injection, intravitreal injection, sustained-release device implantation, and ocular drug electrophoresis. Systemic administration entails oral and intravenous injections, among others.

## Topical GCS

### Topical glucocorticoid eye drops

Primarily utilized for managing anterior uveitis and episcleritis in the context of Behçet's Syndrome, topical glucocorticoid eye drops demonstrate considerable efficacy [30]. However, they do not generally yield substantial results for intermediate, posterior, or panuveitis due to their limited penetration into the deeper parts of the eye. In some cases, aggressive topical therapy may be considered as an adjunctive treatment to enhance therapeutic outcomes for intermediate uveitis, although more potent routes of steroid administration, such as subtenon injections of triamcinolone, remain the primary treatment options. Nonetheless, anterior uveitis occurs less frequently in BS patients. Mydriatic eye drops are often employed concomitantly with GCS to alleviate pain resulting from ciliary muscle spasms and prevent posterior synechiae. In cases of BU involving the anterior segment of the eye, 0.1% dexamethasone eye drops are routinely administered 4–6 times daily. For severe acute uveitis, the frequency of administration may be judiciously increased, with dosing intervals ranging from every 15 min to 1 h, and subsequently reduced or replaced with a less potent glucocorticoid eye drop once inflammation is controlled. Glucocorticoid eye drops, listed in ascending order of corneal penetration capacity, include dexamethasone, prednisolone, fluorometholone, and medrysone. Generally, heightened corneal penetration ability and concentration of glucocorticoid eye drops correlate with augmented anti-inflammatory effects; however, this also increases the likelihood of steroid-related adverse effects.

GCS eye drop usage may precipitate several side effects, such as elevated intraocular pressure (steroid-induced glaucoma), posterior subcapsular cataract, corneal epithelial damage, and herpes simplex viral keratitis. Consequently, patients with corneal lesions, including viral keratitis, fungal keratitis, and corneal ulcers, should refrain from utilizing GCS eye drops.

### Periocular injection

Primarily employed for severe unilateral BU and/or macular edema attributable to BS [31, 32], periocular injections encompass subconjunctival, sub-Tenon, retrobulbar, and peribulbar techniques. Customarily, 40 mg of triamcinolone or 40 mg of methylprednisolone is administered via sub-Tenon or orbital delivery. In cases of acute severe BU, subconjunctival or retrobulbar injections of 2.5–5 mg dexamethasone, administered once daily for a total of 1–3 injections, can be employed to alleviate symptoms before transitioning to long-acting agents such as methylprednisolone [33]. A study by Leder et al. [34] demonstrated that periocular triamcinolone injections significantly ameliorated macular edema in 53% of eyes after one month of follow-up in 126 patients with non-infectious uveitis.

Nevertheless, periocular injections may induce complications such as orbital infection, retrobulbar hemorrhage, and globe perforation, necessitating performance by seasoned ophthalmologists. Complications arising from periocular injections can be bifurcated into those stemming from GCS drugs and those originating from the injection procedure itself. GCS drug side effects parallel those of topical formulations and may encompass steroid-induced glaucoma, cataracts, and corneal epithelial damage. Additionally, the injection procedure may provoke severe complications, including retinal damage, retinal artery occlusion, scarring at the injection site, and bleeding.

### Intravitreal GCS injection 

Intravitreal GCS injections offer superior and consistent local drug concentrations within the eye compared to eye drops and periocular injections. This enhanced penetration into the posterior segment allows for smaller doses with increased efficacy and minimal systemic side effects. As a result, intravitreal GCS injections are suitable treatment options for refractory BU, particularly those with unresponsive cystoid macular edema [35]. Common formulations include triamcinolone acetonide, sustained-release dexamethasone implants, and fluocinolone acetonide intravitreal implants [35].

Tuncer and colleagues administered intravitreal triamcinolone acetonide (IVTA) injections as a complementary therapy in an era when anti-TNF monoclonal antibodies had not yet achieved widespread utilization for the management of Behçet's Uveitis (BU). Their intervention, delivered to a cohort of 15 intractable cases of Behçet's uveitis, led to the complete resolution of inflammation and an enhancement in the best-corrected visual acuity [32]. However, during a 28-month follow-up, 22% experienced relapses and over half developed complications, including elevated intraocular pressure and cataracts. Atmaca et al. also found IVTA injections effective for treating BU-associated macular edema, but also observed increased intraocular pressure in 6 of 10 eyes (60%) post-IVTA injection, with one eye requiring glaucoma surgery [36]. Four eyes (40%) developed cataracts, and two eyes (20%) underwent cataract surgery. Park et al. conducted a retrospective study on 49 severe Behçet's uveitis patients unresponsive to systemic immunosuppressive therapy and treated with IVTA [37]. Over a follow-up exceeding 24 months, mean best-corrected visual acuity improved, but 60% relapsed within 12 months, and cataract surgery probability increased over time. In 40.8% of patients, intraocular pressure exceeded 21 mmHg.

These studies suggest that while intravitreal GCS injections can control acute-phase inflammation in BU, the majority of patients still experience relapses after six months, and the incidence of steroid-related complications remains high. Common complications include elevated intraocular pressure (steroid-induced glaucoma) and concurrent cataract formation (typically posterior capsule opacity). Consequently, experts recommend intravitreal GCS injections as adjunctive treatments to systemic immunomodulatory therapy [38]. However, due to steroid-related side effects and injection-associated complications, repeated injections are not advised.

In summary, intravitreal GCS injections offer enhanced localized drug concentrations in the eye for refractory BU, particularly in cases with cystoid macular edema. This treatment modality, superior to eye drops and periocular injections, improves posterior segment penetration with smaller doses and minimal systemic adverse reactions. Studies demonstrate intravitreal GCS injections' efficacy in controlling acute-phase inflammation and improving best-corrected visual acuity. However, many patients experience relapses after six months, and steroid-related complications, such as elevated intraocular pressure and cataract formation, remain prevalent. Consequently, experts recommend intravitreal GCS injections as an adjunctive treatment to systemic immunomodulatory therapy while discouraging repeated injections due to potential adverse effects and complications.

### GCS sustained-release device

In comparison to intravitreal glucocorticosteroid (GCS) injections, the implantation of GCS sustained-release devices within the vitreous cavity provides a more consistent and prolonged effective drug concentration in the eye. This not only helps prevent inflammation recurrence and achieve long-term remission but also minimizes drug- and injection-related adverse effects [39]. Currently, fluocinolone acetonide and dexamethasone represent the primary sustained-release formulations for intravitreal GCS implantation therapy. In the USA, the FDA has approved a 0.59 mg fluocinolone acetonide implant for treating chronic non-infectious posterior uveitis, offering sustained drug delivery for over 30 months [39].

In a multicenter randomized controlled trial, Pavesio et al. compared the efficacy of 0.59 mg fluocinolone acetonide implantation therapy to traditional therapy, demonstrating that the former significantly lowered uveitis recurrence rates without inducing systemic steroid-related complications, albeit with the potential for local complications such as elevated intraocular pressure and cataracts [39]. Subsequently, Ahmad et al. proposed a combination procedure, incorporating fluocinolone acetonide implantation, Ahmed valves, ciliary body flat tube, cataract extraction, and vitrectomy to minimize postoperative adverse reactions and improve patient prognosis [40]. In 2014, Eun et al. utilized intravitreal fluocinolone acetonide implant therapy on seven patients (eight eyes) with refractory Behçet's uveitis (BU), with an average age of 35.3 years and a mean follow-up of 47.8 months. Following the procedure, the visual acuity of six eyes (75%) improved by three lines or more, five patients discontinued all systemic medications, and six eyes (75%) experienced a postoperative intraocular pressure peak above 30 mmHg, with five patients requiring glaucoma shunt surgery for intraocular pressure management. The results indicated that this therapy effectively controlled refractory BU, with the primary complication being elevated intraocular pressure, necessitating vigilance for potential infection [41]. In 2019, the POINT trial, a multicenter, randomized clinical study, found that both intravitreal triamcinolone acetonide (ITA) and the intravitreal dexamethasone implant (IDI) offered superior efficacy compared to periocular triamcinolone acetonide (PTA) in treating uveitic macular edema, signified by greater reductions in central subfield thickness and better visual acuity improvements, albeit with a modestly increased risk of intraocular pressure elevation. However, there was no significant therapeutic distinction between ITA and IDI, and their intraocular pressure risk profiles were comparable [42]. In 2020, a phase 3 study investigated the long-term efficacy and safety of a 0.2 μg/day fluocinolone acetonide insert (Fai) in treating noninfectious uveitis of the posterior segment (NIU-PS). The findings indicate that over 36 months, the Fai treatment significantly reduced uveitis recurrence rates, extended recurrence-free periods, decreased the number of recurrences per eye, reduced the need for adjunctive therapies, and maintained comparable intraocular pressure to the sham treatment, despite an increased need for cataract surgery, thereby demonstrating an acceptable side-effect profile [43]. In 2021, the seven-year outcomes of the Multicenter Uveitis Steroid Treatment (MUST) trial, comprising 248 eyes from 177 participants with uveitic macular edema (ME), revealed a preference for systemic treatment over Fluocinolone acetonide implants. The trial determined that 94% of eyes experienced resolution of ME at some point during this period, yet relapse occurred in 43% of resolved cases, implicating the significant role of managing inflammation and achieving ME resolution for visual acuity enhancement [44]. The FDA-approved suprachoroidal microinjection of triamcinolone suspension (CLS-TA) has shown significant efficacy in treating uveitic macular edema (UME), as indicated by the PEACHTREE phase 3 clinical trial. In patients not undergoing concurrent systemic corticosteroid or steroid-sparing therapy (ST), CLS-TA demonstrated a mean best corrected visual acuity (BCVA) increase of + 15.6 letters and a mean central subfield thickness (CST) reduction of − 169.8 µm, significantly outperforming controls. Notably, the therapeutic benefit was clinically meaningful for all UME patients, irrespective of additional ST use, and no serious treatment-related adverse events were reported [45].

In 2013, Lightman et al. published a clinical study involving 153 patients with non-infectious uveitis, demonstrating that intravitreal implantation of a 0.7 mg dexamethasone sustained-release device significantly enhanced best-corrected visual acuity after eight weeks, with the therapeutic effect persisting for 26 weeks [46]. In 2015, Coskun et al. conducted a retrospective analysis of the medical records of 12 patients (17 eyes) who received intravitreal dexamethasone (DEX) implants for active BU. The study revealed that a single intravitreal injection of DEX resulted in remission lasting an average of 6.9 months (range 3–12) in 17 eyes, with no relapse in three eyes (18%). Only three eyes (18%) developed elevated intraocular pressure requiring treatment, and no eyes necessitated glaucoma surgery. Among the 13 eyes with crystalline lenses, four eyes developed posterior subcapsular opacities, but no eyes required cataract surgery [47]. A 2017 prospective study by Fabiani et al. examined the efficacy and safety of intravitreal dexamethasone injection in five Behçet's Syndrome (BS) patients with macular edema and retinal vasculitis. The study found significant improvements in mean BCVA and CMT at each follow-up visit, with a mean improvement of 0.26 ± 0.18 lines and 198.80 ± 80.08 µm, respectively, after six months of treatment. The treatment was also effective in resolving all cases of retinal vasculitis. However, one eye experienced elevated intraocular pressure during treatment, and another eye developed significant clinical lens opacity at the six-month follow-up. The authors concluded that intravitreal dexamethasone injection can serve as an adjunctive therapy to support systemic immunomodulatory drug treatment for BU uveitis and inflammatory macular edema. [48].

In summary, intravitreal GCS sustained-release devices provide stable, long-lasting drug concentrations in the eye compared to injections, reducing inflammation recurrence, achieving long-term remission, and minimizing drug-related adverse reactions. Fluocinolone acetonide and dexamethasone are primary sustained-release formulations, with the former FDA-approved for chronic non-infectious posterior uveitis treatment. Studies indicate that fluocinolone acetonide implantation effectively reduces uveitis recurrence rates without systemic steroid-related complications, though local complications like elevated intraocular pressure and cataracts may arise. Combination procedures have been proposed to minimize postoperative adverse reactions and improve prognosis. Similarly, dexamethasone sustained-release device implantation significantly improves best-corrected visual acuity with therapeutic effects lasting up to 26 weeks. Intravitreal dexamethasone implantation has been found to be a safe and effective treatment for Behçet's uveitis and inflammatory macular edema, serving as an adjunctive therapy to systemic immunomodulatory drug treatment.

## Systemic GCS therapy

In instances of BU involving intermediate uveitis, posterior uveitis, and macular edema, systemic glucocorticoid therapy ought to be contemplated, particularly for severe retinal vasculitis and retinal vasculitis induced by BS. Generally, GCS can attain favorable therapeutic outcomes through oral administration and intravenous drip, rendering intravenous administration largely superfluous, particularly to circumvent high-dose steroid pulse therapy utilization.

### Oral administration

Prednisone, a commonly employed GCS medication for treating BU, is typically administered at an initial dose of 1–2 mg/(kg d) in the morning for a duration of 1–2 weeks [38, 49]. As the patient's condition ameliorates, the dose is progressively tapered by 5–10 mg per week. Upon reaching a dose of 30 mg/d, it should be gradually reduced by 2.5–5 mg every 1–2 weeks. During the chronic inflammation phase, the standard maintenance dose is 15–20 mg/d. Subsequent to the maintenance stage, the dose ought to be incrementally decreased to gradually restore adrenal cortical function.

### Intravenous infusion

Intravenous infusion of GCS is generally not advised for BU treatment, as oral administration rapidly attains sufficient drug concentration, minimizing patient discomfort and enhancing compliance. Nevertheless, for patients with acute severe vision-threatening BU, intravenous infusion of methylprednisolone exhibits a swifter onset of action compared to oral GCS, reaching peak blood concentration within 15 min and maintaining a duration of action between 12 and 36 h [50, 51]. Multiple studies have substantiated that high-dose methylprednisolone intravenous pulse therapy can expeditiously control inflammatory reactions in severe BU patients and ameliorate vision [52–54]. The 2018 BS treatment guidelines from the European League Against Rheumatism (EULAR) endorse methylprednisolone intravenous pulse therapy (1 g/d for three days) for BU patients with acute severe vision-threatening symptoms, succeeded by an oral treatment regimen [55]. Furthermore, when systemic high-dose corticosteroid therapy is contraindicated, alternative treatments such as infliximab infusions or interferon monotherapy are recommended for managing severe BU attacks, offering potential therapeutic avenues [55]. Systemic corticosteroids should be administered once in the morning between 7–8 am, coinciding with the physiological peak in plasma cortisol concentration, which subsequently declines from morning to midnight and gradually ascends from midnight to the following morning. Administering GCS in the morning effectively emulates physiological cortisol fluctuation patterns, optimizing efficacy while minimizing adverse reactions [56].

Systemic GCS usage may result in various side effects, including gastrointestinal ulcers, hypertension, diabetes, Cushing's syndrome, and osteoporosis [49, 57]. Furthermore, some studies indicate that long-term GCS administration in BU patients does not appreciably improve their long-term visual prognosis [58, 59]. Consequently, protracted high-dose GCS usage is not advised for BU treatment and should be gradually tapered and combined with other immunosuppressive drugs and biologics to mitigate adverse reactions [32, 55]. In addition, adjunctive periocular or intravitreal GCS injection may be contemplated as a treatment option [31, 32].

## Discussion

The management of BU poses a significant challenge for clinicians due to its recurrent and sight-threatening nature. GCS play a crucial role in the management of Behçet's Uveitis (BU), offering rapid control of intraocular inflammation and mitigating ocular damage. This review highlights the different GCS administration methods, including local injections, sustained-release devices, and systemic therapy. Each method presents its unique advantages and potential drawbacks, emphasizing the need for a tailored approach based on the patient's clinical presentation, systemic conditions, and financial status.

Local GCS injections, particularly periocular and intravitreal, have been shown to provide short-term relief and effectively control inflammation. However, they may be associated with complications such as elevated intraocular pressure and cataract development. GCS sustained-release devices, utilizing fluocinolone acetonide and dexamethasone formulations, offer longer-lasting drug concentrations within the eye, reducing inflammation recurrence and minimizing adverse reactions. Nevertheless, local complications may still arise, necessitating additional procedures to optimize patient outcomes.

Systemic GCS therapy remains an essential tool in managing severe retinal vasculitis and cases involving intermediate uveitis, posterior uveitis, and macular edema. While oral administration is the preferred method, intravenous infusion may be warranted in acute severe vision-threatening BU cases. However, long-term, high-dose GCS use can result in numerous adverse effects and may not significantly improve long-term visual prognosis for BU patients. Consequently, GCS should be combined with other immunosuppressive drugs or biological agents to minimize GCS dosage and reduce the recurrence rate.

## Conclusion

GCS have been indispensable in the management of Behçet's Uveitis, providing rapid control of intraocular inflammation and minimizing ocular damage. However, the potential adverse effects associated with long-term, high-dose GCS use necessitate a cautious approach. The selection of an appropriate GCS regimen should consider the patient's clinical presentation, systemic conditions, and financial status. Combining GCS therapy with immunosuppressive drugs and biological agents can reduce GCS dosage, decrease the recurrence rate, and facilitate long-term remission of uveitis, ultimately improving the overall prognosis for BU patients.

Intravitreal and sustained-release GCS formulations have emerged as valuable additions to the therapeutic armamentarium, reducing systemic exposure and adverse effects. Nevertheless, the administration of GCS in BU management should be carefully balanced against their potential adverse effects, and combination therapy should be optimized to achieve the best therapeutic outcomes. Further research is warranted to elucidate the ideal combination therapy for BU patients, aiming to attain long-term remission of uveitis and improve visual outcomes.

## Data Availability

All data generated or analyzed during this study are included in this published article.
